# COVID-19 in pediatrics: Genetic susceptibility

**DOI:** 10.3389/fgene.2022.928466

**Published:** 2022-08-16

**Authors:** Joseph T. Glessner, Xiao Chang, Frank Mentch, Huiqi Qu, Debra J. Abrams, Alexandria Thomas, Patrick M. A. Sleiman, Hakon Hakonarson

**Affiliations:** ^1^ Children’s Hospital of Philadelphia, Philadelphia, PA, United States; ^2^ Perelman School of Medicine, University of Pennsylvania, Philadelphia, PA, United States

**Keywords:** GWAS, genome-wide association study, diverse populations, pediatrics, statistical genetics and genomics, COVID-19

## Abstract

The uptick in SARS-CoV-2 infection has resulted in a worldwide COVID-19 pandemic, which has created troublesome health and economic problems. We performed case–control meta-analyses in both African and European ethnicity COVID-19 disease cases based on laboratory test and phenotypic criteria. The cases had laboratory-confirmed SARS-CoV-2 infection. We uniquely investigated COVID infection genetics in a pediatric population. Our cohort has a large African ancestry component, also unique to our study. We tested for genetic variant association in 498 cases vs. 1,533 controls of African ancestry and 271 cases vs. 855 controls of European ancestry. We acknowledge that the sample size is relatively small, owing to the low prevalence of COVID infection among pediatric individuals. COVID-19 cases averaged 13 years of age. Pediatric genetic studies enhance the ability to detect genetic associations with a limited possible environment impact. Our findings support the notion that some genetic variants, most notably at the SEMA6D, FMN1, ACTN1, PDS5B, NFIA, ADGRL3, MMP27, TENM3, SPRY4, MNS1, and RSU1 loci, play a role in COVID-19 infection susceptibility. The pediatric cohort also shows nominal replication of previously reported adult study results: CCR9, CXCR6, FYCO1, LZTFL1, TDGF1, CCR1, CCR2, CCR3, CCR5, MAPT-AS1, and IFNAR2 gene variants. Reviewing the biological roles of genes implicated here, NFIA looks to be the most interesting as it binds to a palindromic sequence observed in both viral and cellular promoters and in the adenovirus type 2 origin of replication.

## Introduction

The ongoing coronavirus disease 2019 (COVID-19) pandemic has posed an extraordinary threat to global public health. COVID-19 is caused by the infection of the severe acute respiratory syndrome coronavirus 2 (SARS-CoV-2) (Wu and McGoogan, 2020). SARS-COV-2 is not as virulent as severe acute respiratory syndrome (SARS), and a large number of patients are asymptomatic or suffer only mild symptoms (Bai et al., 2020). The first genome-wide association study (GWAS) of COVID-19 reported two genomic loci associated with severe COVID-19, indicating a strong genetic influence on the severity of COVID-19 (Severe Covid et al., 2020). COVID-19 Host Genetics Initiative performed the largest GWAS in adults to date including 49,562 patients from 46 studies across 19 countries (Initiative, 2020; Kousathanas et al., 2022). They reported 13 genome-wide significant loci that are associated with SARS-CoV-2 infection or severe manifestations of COVID-19.

To date, a number of GWASs on COVID-19 have been reported (Initiative, 2020; Severe Covid et al., 2020; Hu et al., 2021a; Kosmicki et al., 2021a; Pairo-Castineira et al., 2021a; Shelton et al., 2021a; Dubé et al., 2021; Ma et al., 2021; Mousa et al., 2021; Patrick et al., 2021; Peloso et al., 2021; Chamnanphon et al., 2022; Horowitz et al., 2022; Kousathanas et al., 2022; Roberts et al., 2022). The research subjects included European (Initiative, 2020; Severe Covid et al., 2020; Hu et al., 2021a; Kosmicki et al., 2021a; Pairo-Castineira et al., 2021a; Shelton et al., 2021a; Dubé et al., 2021; Ma et al., 2021; Mousa et al., 2021; Patrick et al., 2021; Peloso et al., 2021; Horowitz et al., 2022; Kousathanas et al., 2022; Roberts et al., 2022), African (Kosmicki et al., 2021a; Shelton et al., 2021a; Peloso et al., 2021; Horowitz et al., 2022), East Asian (Mousa et al., 2021; Horowitz et al., 2022), South Asian (Kosmicki et al., 2021a; Mousa et al., 2021; Chamnanphon et al., 2022; Horowitz et al., 2022), and Latin American (Shelton et al., 2021a; Horowitz et al., 2022) populations. The reported studies were all performed on adult populations. In contrast to adults, most of the children infected with COVID-19 presented only mild or moderate symptoms (De Souza et al., 2020), suggesting that different genetic mechanisms from adults may exist. As observed in the GWAS on asthma, 20% of susceptibility loci are pediatric-specific (Ferreira et al., 2019). Due to the gene–environment interaction, some genetic factors may affect sensitivity to environmental factors and vice versa (D’amato et al., 2005). In addition, environmental exposures change over years of life. To date, GWAS of COVID-19 has not been conducted on pediatric populations.

Here, we developed sensitive phenotyping query methods and matched suitable samples to genotyping data pre-QC and post-QC (Table 1). Variants quality controlled with an allele frequency >1%, SNP call rate genotype missingness <0.05, Hardy–Weinberg equilibrium deviation *p*-value > 1e-6, and imputation quality R2 > 0.3 were further assessed in African and European studies. Despite many active studies, the genetics impacting SARS-CoV-2 infection risk and progression severity remains poorly understood. The SNP-based associations were refined based on peaks of significance for contiguous SNPs and linkage disequilibrium (LD) of top significant SNP regions. Further work on larger cohorts is needed to better understand which traits (disease, health, and neuropsychiatric phenotypes) are genetically correlated and potentially causally associated with the infection of SARS-CoV-2. Tremendous worldwide COVID-19 genotype aggregation efforts have launched sample sizes of 49,562 patients with COVID-19 and 2 million controls (Niemi et al., 2021). PLINK23 (Purcell et al., 2007) software was leveraged for efficient quality filtering, statistical association, and review of results.

**TABLE 1:** COVID-19 pediatric case–control cohorts analyzed.

	Total queried phenotype	Total (pre-QC)	EUR (pre-QC)	AFR (pre-QC)	Total (post-QC)	EUR (post-QC)	AFR (post-QC)
Case	994	841	286	555	769	271	498
Control	2965	2490	873	1617	2388	855	1533

## Results

To limit the chance of spurious associations, implicated disease phenotypes associated with SNPs in LD (r2 > 0.8) with the top significant COVID-19 variants were reviewed. The inclusion of pediatric cases and controls from both European and African ancestries demonstrates the value of including data from diverse populations for characterizing genetic associations. Environmental, clinical, and social factors contribute to exposure and severity of COVID-19 (Docherty et al., 2020; Zhou et al., 2020) with host genetics also playing an important role. Here, we show genome-wide association meta-analysis results that consist of 498 pediatric cases vs. 1,533 controls of African ancestry and 271 pediatric cases vs. 855 controls of European ancestry (Tables 2, 3).

**TABLE 2:** Replication of previous findings.

Chr:Start–end (hg38/GRCh38) (*P* < 0.05)	Gene	Cohort	Lead SNP	P (E)	OR	Broad cohort	Broad lead SNP	Broad P	Broad beta
3:45848457–45976785	*CCR9*, *CXCR6*, *FYCO1*, and *LZTFL1*	EUR	3:45961470:T:C	2.55–3	0.729	AFR + EUR META	3:45848457:C:T	4.25E-81	0.588
3:46610496–46610496	*TDGF1*	EUR	3:46610496:A:G	5.02–3	3.006	EUR META	3:46610496:A:G	4.99E-8	0.427
3:46108627–46374725	*CCR1*, *CCR2*, *CCR3*, and *CCR5*	EUR	3:46108627:C:T	5.84–3	0.750	AFR + EUR META	3:46231218:A:C	3.47E-20	0.304
17:45880713–45880713	*MAPT-AS1*	AFR	17:45880713:C:T	5.92–3	1.331	AFR META	17:45880713:C:T	2.68E-8	-0.127
21:33238182–33238182	*IFNAR2*	EUR	21:33238182:T:C	3.36–2	1.244	EUR META	21:33238182:T:C	1.01E-11	0.128

**TABLE 3:** Novel findings of this study.

Chr:Start–end (hg38/GRCh38) (*P* < 5e-5)	Gene	AFR_SNP	EUR_SNP	META_SNP	AFR_P	EUR_P	META_P	AFR OR	EUR OR	Meta OR
15:47504866–47504920	*SEMA6D*	15:47504920:T:TG	NA	15:47504866:C:G	9.80E-06	NA	NA	2.300	NA	NA
15:33036296–33036318	*FMN1*	15:33036296:T:C	15:33036296:T:C	15:33036296:T:C	3.17E-04	1.07E-02	1.04E-05	1.337	1.305	1.325
14:68910449–68910548	*ACTN1*	14:68910548:A:G	14:68910449:T:C	14:68910548:A:G	2.61E-01	1.09E-05	1.09E-02	1.169	5.140	1.389
13:32665329–32665331	*PDS5B*	13:32665331:T:C	NA	13:32665329:A:C	1.19E-05	NA	NA	2.349	NA	NA
1:61414689–61414750	*NFIA*	1:61414689:T:C	1:61414750:A:G	1:61414689:T:C	1.80E-05	4.63E-01	1.24E-03	0.692	1.103	0.792
4:61421195–61421214	*ADGRL3*	4:61421195:T:A	NA	4:61421195:T:A	2.14E-05	NA	NA	2.783	NA	NA
11:102697419–102697493	*MMP27*	11:102697419:G:A	11:102697419:G:A	11:102697419:G:A	1.90E-03	3.66E-03	2.23E-05	1.299	1.357	1.321
4:182739578–182739648	*TENM3*	NA	4:182739648:G:A	4:182739578:C:A	NA	2.43E-05	NA	NA	1.597	NA
5:142320157–142320171	*SPRY4*	5:142320157:T:C	NA	5:142320157:T:C	3.08E-05	NA	NA	1.999	NA	NA
15:56458970–56459025	*MNS1*	15:56458970:A:G	15:56458970:A:G	15:56458970:A:G	2.05E-03	5.58E-03	3.39E-05	0.739	0.736	0.738
10:16597265–16603587	*RSU1*	10:16597265:G:A	NA	10:16597265:G:A	3.76E-05	NA	NA	2.193	NA	NA

**FIGURE 1:**
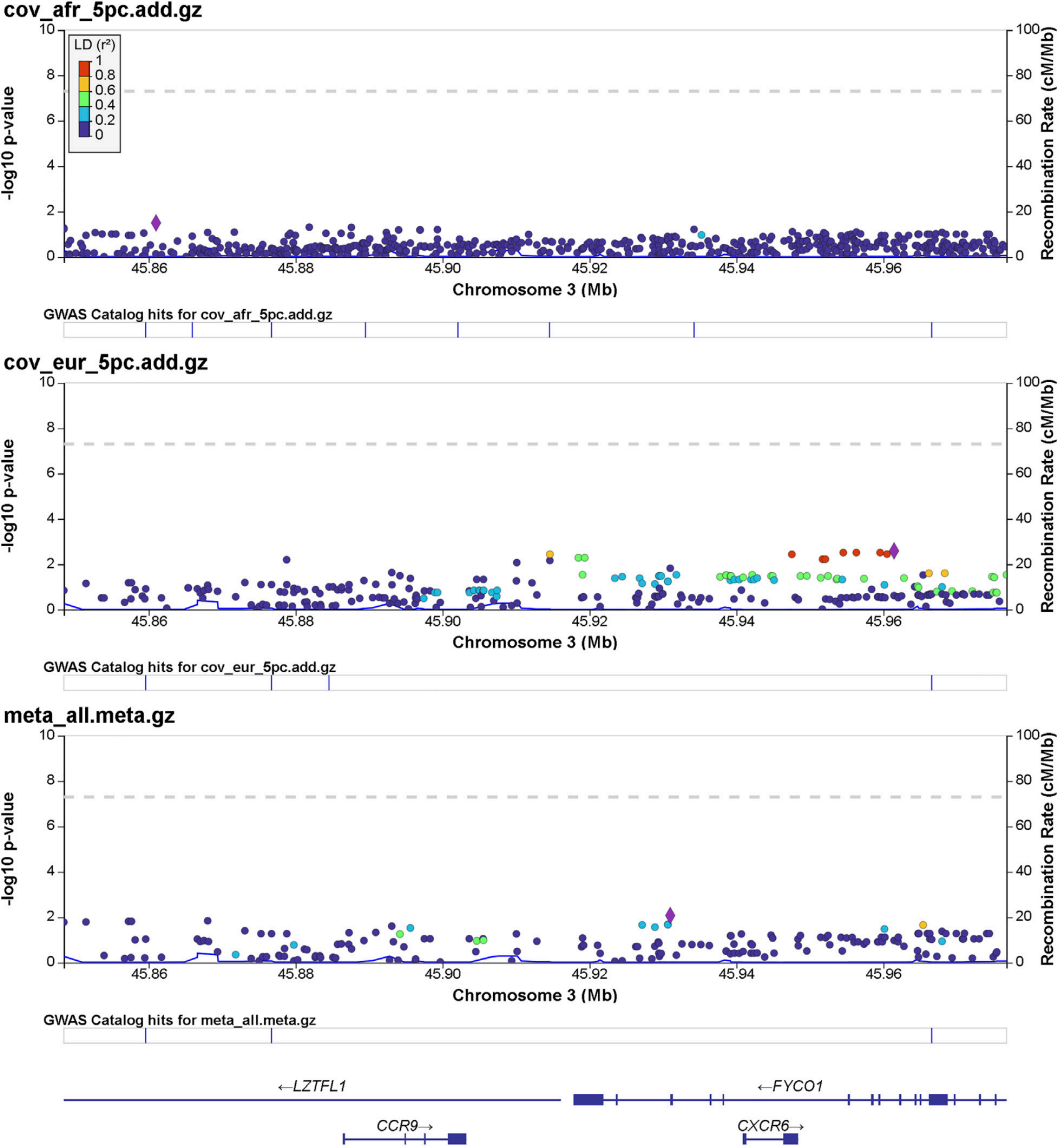
Regional significance plot of the 3p21.31 region by LocusZoom (Pruim et al., 2010). The genes *CCR9*, *CXCR6*, *FYCO1*, and *LZTFL1* are included in this region. The peak of significance is from the SNP 3:45961470:T:C (rs1601867) at the intronic region of *FYCO1*.

**FIGURE 2:**
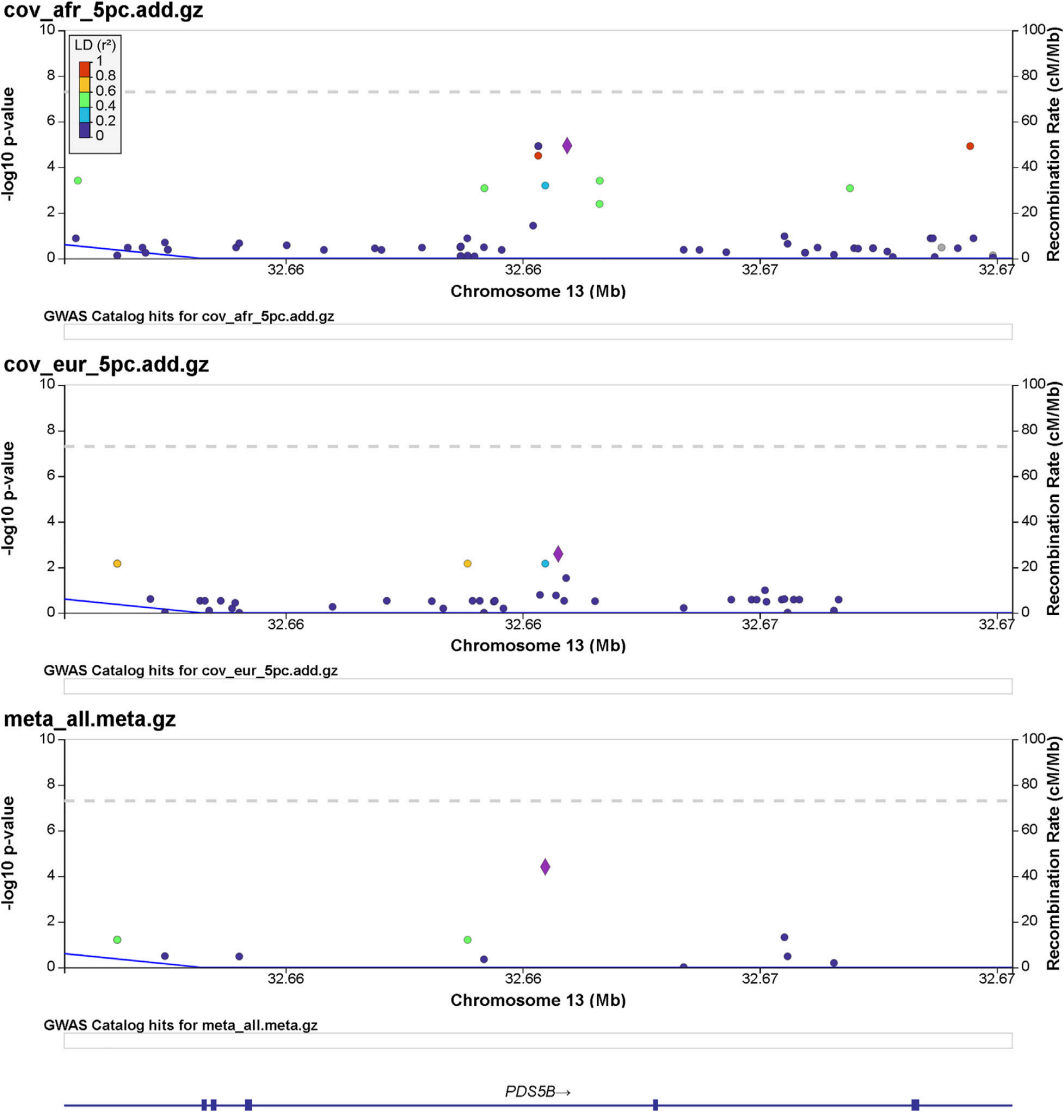
Regional significance plot of the 13q13.1 region by LocusZoom (Pruim et al., 2010). The PDS5 cohesin-associated factor B gene (*PDS5B*) maps to this region. The peak of significance by the meta-analysis is from the SNP 13:32665329:A:C (rs144965594) at the intronic region of *PDS5B*.

Details of genomic loci and observed significance are provided in LocusZoom (Pruim et al., 2010) plots (Figures 1, 2). Replicating a previously reported study (Roberts et al., 2020), a top significant locus in our results was within the 3p21.31 region associated with SARS-CoV-2 infection susceptibility (Table 2). We referenced cis-protein QTLs (Sun et al., 2018) to more deeply characterize the top significant loci. We used the European and African reference panel from TOPMed and the 1000 Genomes Project (Abecasis et al., 2010) to show LD between genetic variants. Genetic variants underlying COVID-19 susceptibility holds the potential to glean models of disease biology for therapeutic development, to extend new prevention and treatment options beyond the recent release of vaccines. Some of the most significantly associated SNPs (Table 2) overlap previously confirmed genetic associations as mentioned previously (David et al., 2020; Pairo-Castineira et al., 2021b).

## Discussion

Among the reported genes by the previous GWASs (Initiative, 2020; Severe Covid et al., 2020; Hu et al., 2021a; Kosmicki et al., 2021a; Pairo-Castineira et al., 2021a; Shelton et al., 2021a; Dubé et al., 2021; Ma et al., 2021; Mousa et al., 2021; Patrick et al., 2021; Peloso et al., 2021; Chamnanphon et al., 2022; Horowitz et al., 2022; Kousathanas et al., 2022; Roberts et al., 2022), interestingly, genes related to cytokine receptor activity (GO:0004896) are significantly enriched (FDR-corrected *p* = 0.017) by gene-set enrichment analysis using the WebGestalt (WEB-based Gene SeT AnaLysis Toolkit) web tool (Wang et al., 2013). The genes include C-C motif chemokine receptor 1 (*CCR1*), C-C motif chemokine receptor 3 (*CCR3*), C-C motif chemokine receptor 9 (*CCR9*), C-X-C motif chemokine receptor 6 (*CXCR6*), interferon alpha and beta receptor subunit 2 (*IFNAR2*), interleukin 10 receptor subunit beta (*IL10RB*), LIF receptor alpha (*LIFR*), and X-C motif chemokine receptor 1 (*XCR1*). As shown in Table 2, the genes related to cytokine receptor activity, including *CCR1*, *CCR3*, *CCR9*, *CXCR6*, and *IFNAR2*, are also identified in this study. Chemokine receptors are G protein-coupled receptors and bind chemokines to mediate cell migration in immune surveillance and inflammation (Allen et al., 2007). *CCR1*, *CCR3*, and *CCR9* encode receptors of the C-C family chemokines with two adjacent N-terminal cysteine residues. There are 28 C-C chemokines and 10 C-C chemokine receptors identified to date (White et al., 2013). CCR1 and CCR3 bind to multiple CC chemokines with critical roles in inflammation (Pakianathan et al., 1997). *CCR9* encodes the receptor of C-C motif chemokine ligand 25 (CCL25), with a role in the development of T cell in thymus (Vicari et al., 1997). CXCR6 has a protein structure close to CCRs and binds to the ligand CXCL16 of the CXC family chemokines with one amino acid between the two N-terminal cysteine residues (Day et al., 2009). CXCR6 may have important roles in T-cell recruitment to the lung in COVID-19 infection, as suggested by its high expression in the lung (Day et al., 2009). *IFNAR2* encodes subunit 2 of the interferon-α/β receptor (IFNAR) (Lutfalla et al., 1995), mediating the roles of type 1 interferons α and β in innate immune response to viral infections (Biron, 1998). In addition to the roles of the cytokines in anti-viral immunity and inflammation (Bartee and McFadden, 2013), these genes may also be involved in cytokine storm in severe COVID-19 (Hu et al., 2021b).

We show here 13 ethnicity-specific and/or meta-analysis variants that pass the top rank and nominal significance threshold (*p* < 5e-5). Several genome-wide association studies investigating case and control samples with many SNP genotypes, which have associated certain SNPs (David et al., 2020; Roberts et al., 2020; Pairo-Castineira et al., 2021b; Shelton et al., 2021b) to COVID-19, have indicated support for several genomic loci associated with COVID-19 susceptibility and severity; the strongest association related to severity is at the 3p21.31 locus (David et al., 2020; Roberts et al., 2020; Kosmicki et al., 2021b; Pairo-Castineira et al., 2021b; Shelton et al., 2021b). Two separate loci in the 3p21.31 region include genes prioritized from different methods and signals.

A number of loci identified in this study have not been reported in the previous GWASs on adults (Initiative, 2020; Severe Covid et al., 2020) (Table 3). Interestingly, five of these loci, i.e., *ACTN1*, *PDS5B*, *SEMA6D*, *SPRY4*, and *TENM3*, have been reported of association with the genetic susceptibility of asthma (Yucesoy et al., 2015; Almoguera et al., 2017; Demenais et al., 2018; Olafsdottir et al., 2020). As reviewed by Adir et al. (2021), asthma may impose important factors related to SARS-CoV-2 infection and disease severity, for e.g., Th2-high inflammation in asthma may reduce the risk of SARS-Cov-2 infection and chronic use of systemic corticosteroids (ICS) is associated with poor outcomes of COVID-19.

Further population sampling and genotyping of COVID-19 and related phenotypes is warranted to further characterize susceptibility, severity, or mortality in the future, guided by Centers for Disease Control enumeration of prior medical conditions linked with COVID-19 severity (CDC, 2021) or traits linked with risk of COVID-19 mortality by OpenSAFELY (Williamson et al., 2020).

### Limitations

This study has limitations. First, the controls were determined based on the records from our EMR data collected in October 2021. The controls might get infected at a later time point. As COVID-19 is an infectious disease, this limitation exits in all COVID GWASs. Second, the sample size is relatively small. Future studies with a larger sample size may identify genetic loci of COVID-19, especially associated with pediatric populations. Third, this study was performed on a unique pediatric cohort of COVID-19. However, we acknowledge that follow-up analyses for the novel loci described in this study are warranted.

## Conclusion

Our analyses report 17 independent genome-wide nominal significance SNPs with neighboring higher than expected *p*-value SNPs (6 were replication of previous findings and 11 were novel findings), defining COVID-19 loci with a threshold of *p* < 5 E-5 (unadjusted for multiple testing). A unique and challenging aspect is variable progression of SARS-CoV-2 infection, ranging from acute to severe clinical presentations of viral pneumonia or acute respiratory distress syndrome (Buitrago-Garcia et al., 2020). Additional cohorts and studies will be needed to effectively leverage biological and clinical yield potential of these genetic associations. We applied covariates including age, sex, and the five first principal components to properly account for these population characteristics in addition to the SNP genotypes. For all 13 loci, we compared the lead variant (strongest association *p*-value) and odds ratios (ORs) for the risk allele across different ethnic groups. Four of the thirteen genome-wide nominal significant loci showed similar trends in SARS-CoV-2 infection (i.e., disease susceptibility). Host-specific genetic variants identified here hold the potential to characterize biological interaction and function, informing therapeutic possibilities, and delineate causal link of risk factors in the environment for SARS-CoV-2 infection and prognosis. These findings indicate a multi-gene and multi-function mechanism to be more fully characterized by future studies.

## Methods

### Subjects

All subjects were recruited using CHOP Institutional Review Board-approved protocols. The SARS-CoV-2 infection-positive group had laboratory-confirmed SARS-CoV-2 infection, electronic health record ICD coding, or was self-reported by a survey, along with the annotation whether symptoms of severity were observed. The Diagnosis and Treatment Protocol for Novel Coronavirus Pneumonia was used to classify illness severity and hospitalization observations (Wei, 2020). Controls were populations based on data of negative SARS-CoV-2 infection and negative COVID-19 status. Genetic-ancestry-matched control individuals for the COVID-19-positive cases were matched with population-based cohorts based on nearest PCA distance. Control individuals were infection-negative based on questionnaire/electronic health record-based database queries.

### Genotyping

Samples were genotyped using the Illumina Infinium BeadChip Global Screening Array (GSA). SNP genotypes and variant allele naming were coordinated to human genome build hg38/GRCh38 and referenced with respect to gnomAD 3.0 genomes (Karczewski et al., 2020) by matching SNPs via variant matching by testing strand flip and allele order switches. To gain additional resolution of genotyping data for these samples, we performed imputation on the TOPMed Imputation Server at https://imputation.biodatacatalyst.nhlbi.nih.gov/.

### African ancestry COVID case–control

A total of 367,556 genetic variants passed filters and quality control and thus were tested for association to COVID-19-infected phenotype individuals. A total of 2,172 individuals (1,017 males and 1,155 females) were included. The total genotyping rate in samples remaining after quality control was 0.997553. The number of individuals who passed filters and QC was 2,031. Among the remaining phenotypes, 498 were cases and 1,533 were controls.

### European ancestry COVID case–control

A total of 486,109 variants were assessed that met filter and QC standards. A total of 1,159 individuals (643 males and 516 females) were included. The total genotyping rate in the remaining samples was 0.998073. Altogether, 486,109 variants and 1,126 individuals passed filters and QC. Among the remaining phenotypes, 271 were cases and 855 were controls.

### African ancestry and European ancestry COVID meta-analysis

A meta-analysis including 14,336,851 variants was processed, and 3,854,317 variants had non-NA *p*-values. Several known clinical factors of the host track closely to disease severity such as older age, being male, and larger body mass index (Docherty et al., 2020), but these factors are not sufficient to model disease severity variability. These findings support prioritizing candidate genes along with future functional characterization to refine the genes. Control samples were chosen based on principal component analysis-driven genetic ancestry-matching samples without known SARS-CoV-2 infection.

### Data analysis

To prioritize candidate gene regions reported in this study, we used both locus-based and similarity-based methods. We report the raw *p*-values and odds ratios for each lead variant with closely adjacent nominal significance variants along with the nearest gene. In an effort to better characterize the biological mechanism of observed variants at each locus, we prioritized candidate genes and referenced knowledge from results from related diseases and traits. The relevant stage of disease from SARS-CoV-2 infection to progression and outcome was a factor considered in the modeling of gene roles in associated loci.

We used PLINK2 (Chang et al., 2015) to apply sample and SNP quality control thresholds, in association with an additive effect model, applying the top five principal components as covariates and conducting meta-analysis.

We conducted GWAS statistical analyses with the tool Scalable and Accurate Implementation of GEneralized (SAIGE) mixed model (Zhou et al., 2018) on all autosomes and chromosome X. Our 17q21.31 replication top finding overlapping MAPT-AS1 (KANSL1 150 kb away) coincides with a deeply studied locus with structural variants including a large megabase recurrent inversion deviating from the reference H1 to the inverted H2 form that has been selected positively in European ancestry persons (Stefansson et al., 2005; Boettger et al., 2012). SAIGE features robust modeling of sample relatedness and case–control count differences. The genetic identity of a person influences viral infection susceptibility and response. We sought to characterize the 13 nominal significant loci for potential to fulfill roles in risk and progression following infection. We used the Cochran’s Q measure (Cochran, 1954; Evangelou and Ioannidis, 2013) using the two analyses effect sizes vs. the meta-analysis effect size (weighted by inverse variance of effect sizes) sum of squared differences.

## Data Availability

The datasets presented in this study can be found in online repositories. The names of the repository/repositories and accession number(s) can be found at: https://www.ebi.ac.uk/gwas/studies/, GCST011074 and GCP000381.
